# *Fasciola* Infection Unexpectedly Found During Cholecystectomy: Review on How to Avoid Increasing Surgery Interventions in Non-human Endemic Areas

**DOI:** 10.1007/s11686-023-00726-6

**Published:** 2023-11-07

**Authors:** Gholamreza Mowlavi, María Dolores Bargues, Faezeh Najafi, Saied Reza Naddaf, Alireza Salehabadi, Amir Kazem Vejdan, Mahboobeh Salimi, Arezoo Fadavi, Zahra Arab-Mazar, Santiago Mas-Coma

**Affiliations:** 1 Department of Medical Parasitology and Mycology, School of Public Health, Tehran University of Medical Sciences, Tehran, Iran; 2 Center for Research of Endemic Parasites of Iran (CREPI), Tehran University of Medical Sciences, Tehran, Iran; 3 Departamento de Parasitología, Facultad de Farmacia, Universidad de Valencia, Av. Vicent Andres Estelles s/n, Burjassot, 46100 Valencia, Spain; 4 CIBER de Enfermedades Infecciosas, Instituto de Salud Carlos IIII, C/ Monforte de Lemos 3-5, Pabellón 11, Planta 0, 28029 Madrid, Spain; 5 Department of Parasitology, Research Center for Emerging and Reemerging Diseases, Pasteur Institute of Iran, Tehran, Iran; 6 Department of Microbiology, Birjand University of Medical Sciences, Birjand, Iran; 7 Department of General Surgery, Imam Reza Hospital, Birjand University of Medical Sciences, Birjand, Iran

**Keywords:** Human fascioliasis, Cholecystectomy, *Fasciola* hybrid, Eastern Iran, Misdiagnosing presentations, Avoiding unnecessary surgery

## Abstract

**Purpose:**

Fascioliasis is caused by *Fasciola hepatica* of almost worldwide distribution and *F. gigantica* in wide regions of Asia and Africa. Their adult stage develops in the biliary canals and gallbladder. Infection follows an initial, 3–4 month long invasive, migratory or acute phase, and a several year-long biliary, chronic or obstructive phase.

**Methods:**

The unexpected finding of a fasciolid inside the gallbladder during a cholecystectomy for obstructive lithiasis suspicion in a patient is reported from an area of Iran where human infection had been never reported before and studies on fascioliasis in livestock are absent.

**Results:**

The fluke obtained was phenotypically classified as *F. hepatica* by morphometry and genotypically as *F. gigantica* by mtDNA *cox*1 fragment sequencing, although with *F. hepatica* scattered mutations in species-differing nucleotide positions. The clinical, radiological, and biological signs observed at the acute and chronic phases often lead to some misdiagnosis. Serological methods may be useful in cases of negative coprology. Diagnostic techniques with insufficient resolution leading to unnecessary invasive interventions are analyzed. The way to avoid unnecessary surgery is described, including analyses to be made, diagnostic tools to be used, and aspects to be considered.

**Conclusion:**

Reaching a correct diagnosis in the confusing presentations avoids procedure delays and unnecessary surgery. A correct drug treatment may be sufficient. Except in extreme pathological presentations, lesions decrease in number and size and finally disappear or calcify after a successful treatment. Finally, the need to increase awareness of physicians about fascioliasis is highlighted, mainly in non-human endemic areas.

## Introduction

Fasciolid flukes are trematodes whose adult stage develops in the biliary canals and gallbladder of humans and animals. Fascioliasis, a disease of medical and veterinary importance, is caused by *Fasciola hepatica* distributed throughout almost the whole world and preferring mild and cold environments, and *F. gigantica*, in warm lowlands of wide regions of Asia and Africa [1]. These different geographical distributions are defined by their respective specific intermediate freshwater snail hosts or vectors of the family Lymnaeidae, assuring fascioliasis transmission in rural areas [2, 3]. Their veterinary importance is related to the losses this disease causes in livestock husbandry [4], mainly ruminants but also pigs, equines, and other herbivorous mammals [1].

Until quite recently it was considered that *F. hepatica* was the cause of human fascioliasis in most of the cases, whereas *F. gigantica* was only rarely involved in human infection. Indeed, most of the human cases reported concern *F. hepatica* [5]. However, recent studies have demonstrated that *F. gigantica* is increasingly involved both in individual patient reports and in human endemic areas in Asia and Africa. In addition, the latter species has recently been proved to be more pathogenic than *F. hepatica*, due to the bigger size of the *F. gigantica* adult stage [6].

Both *Fasciola* species cause a similar disease including two clinical phases [7]:

- An invasive, migratory or acute phase, corresponding to the hatching of the ingested metacercariae at duodenal level, intestinal wall crossing by the juvenile fluke, migration through the abdominal cavity, and penetration into the liver parenchyma up to the biliary canals. This initial 3–4-month long phase usually shows pronounced symptomatology which leads patients to request healthcare and therefore enables for early detection of infection. There are, however, cases in which this phase develops asymptomatically or with mild symptomatology that may be diagnostically confused or undetected because of shortly transitory or insufficient as to require medical attention.
- A biliary, chronic or obstructive phase, including the fluke adult inside the biliary canals and/or gallbladder and which may last many years in humans. Long time considered of pathogenicity and symptomatology lower than the acute phase, present knowledge has demonstrated its pathogenicity and immunosuppression capacity facilitating coinfections by other pathogenic helminths, protozoans, and bacteria.

Fascioliasis is a disease highly influenced by climate and global changes. These presently ongoing phenomena are modifying fascioliasis prevalences and intensities and facilitating the spread of the disease [8], as in other trematodiases [9]. In a wide area of Pakistan, fascioliasis transmission monoseasonality has changed to a higher-infection-risk biseasonality including a peak linked to climate and another peak to man-made irrigation management [10]. The worrying scenario led the World Health Organization to include fascioliasis within the group of foodborne trematodiases among Neglected Tropical Diseases [11].

Present trends indicate that fascioliasis transmission will increase in many areas and will colonize new areas, both in developed and in developing countries [12]. Expected risks suggest higher human infection probabilities in the future, including areas where human infection cases have been reported never before or only very sporadically. The problem for local physicians who will not be aware on this disease because of lack of knowledge of the disease and having never faced a fascioliasis patient is evident.

Here we report one of such cases, in which a fasciolid fluke was unexpectedly found inside the gallbladder during cholecystectomy in eastern Iran. The fasciolid specimen obtained was phenotypically characterized by morphometry and molecularly classified by means of DNA sequencing. Patient infection presentations able to lead to confusion are reviewed. Diagnostic techniques with insufficient resolution leading to unnecessary invasive interventions are analyzed. The way to avoid unnecessary surgery is described, including analyses to be made, diagnostic tools to be used, and aspects to be considered.

## Materials and Methods

### Geographical Origin

**Fig. 1:**
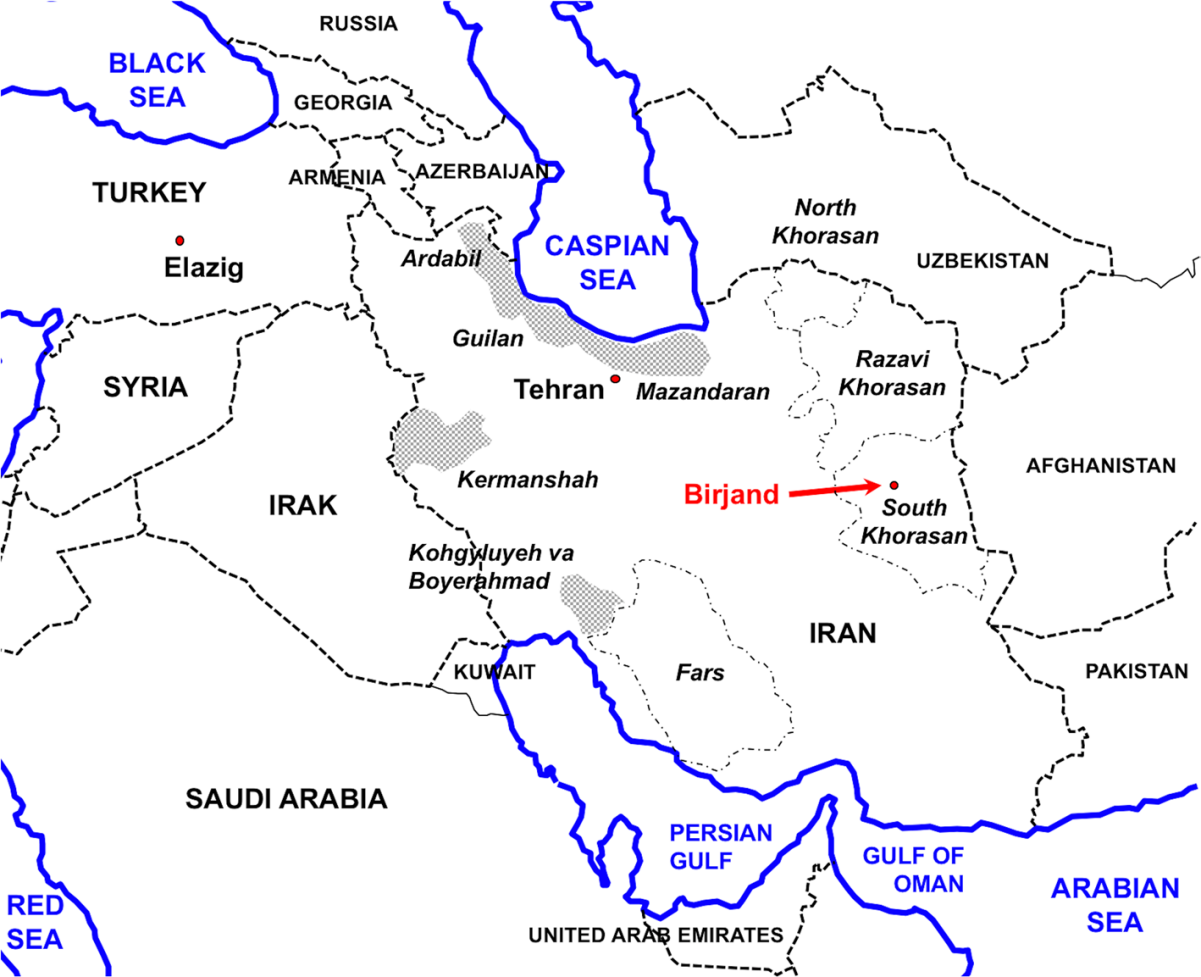
Map showing the locality of Birjand, capital of South Khorasan Province, in eastern Iran, where the infected surgically intervened patient came from. Note: location close to Afghanistan and Pakistan where intermediate fasciolid forms are known; western areas of Iran where human infection has been reported (shaded areas); northeastern provinces of Northern Khorasan and Razavi Khorasan where fascioliasis has been reported in livestock; province of Fars where the only previous Iranian report of an intermediate fasciolid has been reported in animals; and Turkish locality of Elazig where a similar mtDNA *cox*1 sequence has been found

The geographical location of the locality of Birjand, from whose vicinity the patient came from, together with the areas where human infection has been repeatedly reported in Iran, are shown in Fig. 1. Birjand is the capital of South Khorasan Province, in eastern Iran, close to the Afghanistan border and near Pakistan.

### Phenotypic Analysis

The only fasciolid specimen obtained from the patient during surgical intervention and fixed in formalin was phenotypically characterized on the basis of standardized measurements known to be useful for the differentiation of fasciolid species [13, 14]. Standardized measurements were taken using a microscope equipped with a Camera Lucida. The following standardized lineal biometric characters were measured (mm): body length (BL), maximum body width (BW), oral sucker diameter (OS), distance between the oral sucker and the ventral sucker (OS–VS), distance between the ventral sucker and the posterior end of the body (VS–P), distance between the anterior end of the body and the ventral sucker (A–VS), cone length (CL), and cone width (CW). In addition, the following ratios were calculated: BL over BW (BL/BW), and BL over the distance between the VS and P (BL/VS–P).

The results were compared to standard pure populations of *F. hepatica* and *F. gigantica* [14] similar results previously obtained for both species in Iran [15, 16], and also from eastward neighboring Pakistan [17].

A few fasciolid eggs could be found in the container where the surgically extracted worm was fixed and conserved. These eggs were characterized by measuring their length (EL) and maximum width (EW) and subsequent comparison with egg size standards of *F. hepatica* and *F. gigantica* [18].

### Molecular Analysis

#### DNA Extraction

Genomic DNA from the formalin-fixed worm was extracted using high pure PCR template preparation kit (Roche, Germany) following the manufacturer's procedure protocol.

#### PCR Amplification and Sequencing of Cox1 Fragment

In a first approach, we performed a PRC assay to amplify a target region (405 bp) of the cytochrome c oxidase subunit 1 of the mitochondrial DNA (mtDNA *cox*1 gene) using primers FhCO1F (5′-TATGTTTTGATTTTACCCGGG-3′), and FhCO1R (5′-ATGAGCAACCACAAACCATGT-3′) [19]. This assay was unsuccessful. In the second run of amplification, a nested PCR (nPCR) assay was performed using primers designed in this study, namely FgCO1F (5′-GCTATGGCTGCTATAGTATG-3′) and FgCO1R (5′-CCAGTAACCCCACCAATAGT-3′). With these new primers, a fragment of 247 bp could be successfully amplified.

The 25 µl reaction mixture contained 12.5 µl of 2 × red load Taq Master (Jena Bioscience, Korea), 2 µl of each primer, 2 µl of DNA, and 18.5 µl of distilled water for the first amplification round, and 2 µl of 1/80 diluted first-round PCR product for the second amplification round. The PCR procedure in both rounds consisted of 60 s of denaturation at 95 °C, followed by 60 s of annealing at 56 °C and 90 s of extension at 72 °C, for 30 cycles. An initial step of 3 min at 98 °C and a final step of 3 min at 72 °C were included.

Nine microliters of the amplification products were electrophoresed on a 1.2% agarose gel and visualized using ultraviolet light after staining with safe stain. A negative control (2 × red load Taq Master, primers, and distilled water) and one positive control (DNA extracted from *F. hepatica*) were included in each round. Except for positive control, no amplification was detected in the first amplification round. The nested primers, however, successfully induced amplification of a 247 bp PCR product. No amplification was detected in negative control in both rounds.

The nested PCR products were sequenced on both directions by the dideoxy chain termination method using the same amplification nPCR primers.

### Sequence Analysis

Sequence results were edited and analyzed by BioEdit version 7.0.5.3 and the consensus sequence was compared with reference sequences of the fasciolid haplotype collection of the WHO Collaborating Centre of Valencia [1], and with similar sequences available in the GenBank database. Alignments were made using CLUSTALW2 [20] in MEGA version 7 [21], using default settings. Homologies were performed using the BLASTN program from the National Center for Biotechnology Information website (http://www.ncbi.nlm.nih.gov/BLAST).

## Results

### Case Presentation

A 70-year-old man went to a clinic presenting with pain in the abdomen and sternum and moderate jaundice. The patient resided in the vicinity of Birjand, the capital of South Khorasan province, eastern Iran close to the Afghanistan border (Fig. 1). His professional activities were linked to animal husbandry and agriculture in a rural area. In the anamnesis, the patient answered that he had not traveled to other provinces known to be endemic of fascioliasis, thus suggesting a local infection. Although he did not specifically refer to having consumed sylvatic plants, his professional activities related to livestock keeping and vegetable cultivation during his whole life were evidently linked to a high fascioliasis infection risk, because of working close to the main domestic herbivore animal reservoirs of the disease and the management of consumable vegetables needing irrigation.

A sudden discomfort and pain led the patient to the Birjand hospital, because there was no health center nor laboratory in the rural village where he lived. At hospital admission, the patient manifested to suffer from pain in the form of biliary colic, which he described to sometimes last for 2–3 weeks in the form of the right upper quadrant. Such symptoms were improved after 3–4 h with medical and analgesic treatments.

The initial clinical observation was indicating gallbladder obstruction due to the presence of stones. He was, therefore, directly considered a candidate for surgical intervention in the way for cholecystectomy. A diagnosis of long-term lithiasis was further supported by the liver images furnished by ultrasonography (US) suggesting the presence of stones. This correlated well with 8-year history of typical symptoms of gallstone disease.

During the operation, the surgeons observed an alive highly motile fluke inside the gallbladder. The specimen was extracted, preserved in formalin, and subsequently transferred to the Laboratory of Helminthology of the School of Public Health, Tehran University of Medical Sciences for classification. Additional fasciolid egg finding in the transport container confirmed that the patient was in the chronic phase.

Interestingly, in the analysis of liver damage parameters by standard serological methods before the surgical intervention decided after the diagnosis of long-term lithiasis, neither eosinophilia nor clear alterations of other serum biochemical parameters outside of the normal range values were observed. These results were interpreted by considering three main aspects, namely (i) the very low infection burden, as only one liver fluke specimen was found, (ii) the very long infection after a chronicity period of more than 8 years of gallstone disease symptoms, and (iii) the infection being restricted to the gall bladder in the moment of the intervention, a microhabitat within which the unique liver fluke specimen was probably located after long time.

### Morphometric Characterization

**Fig. 2:**
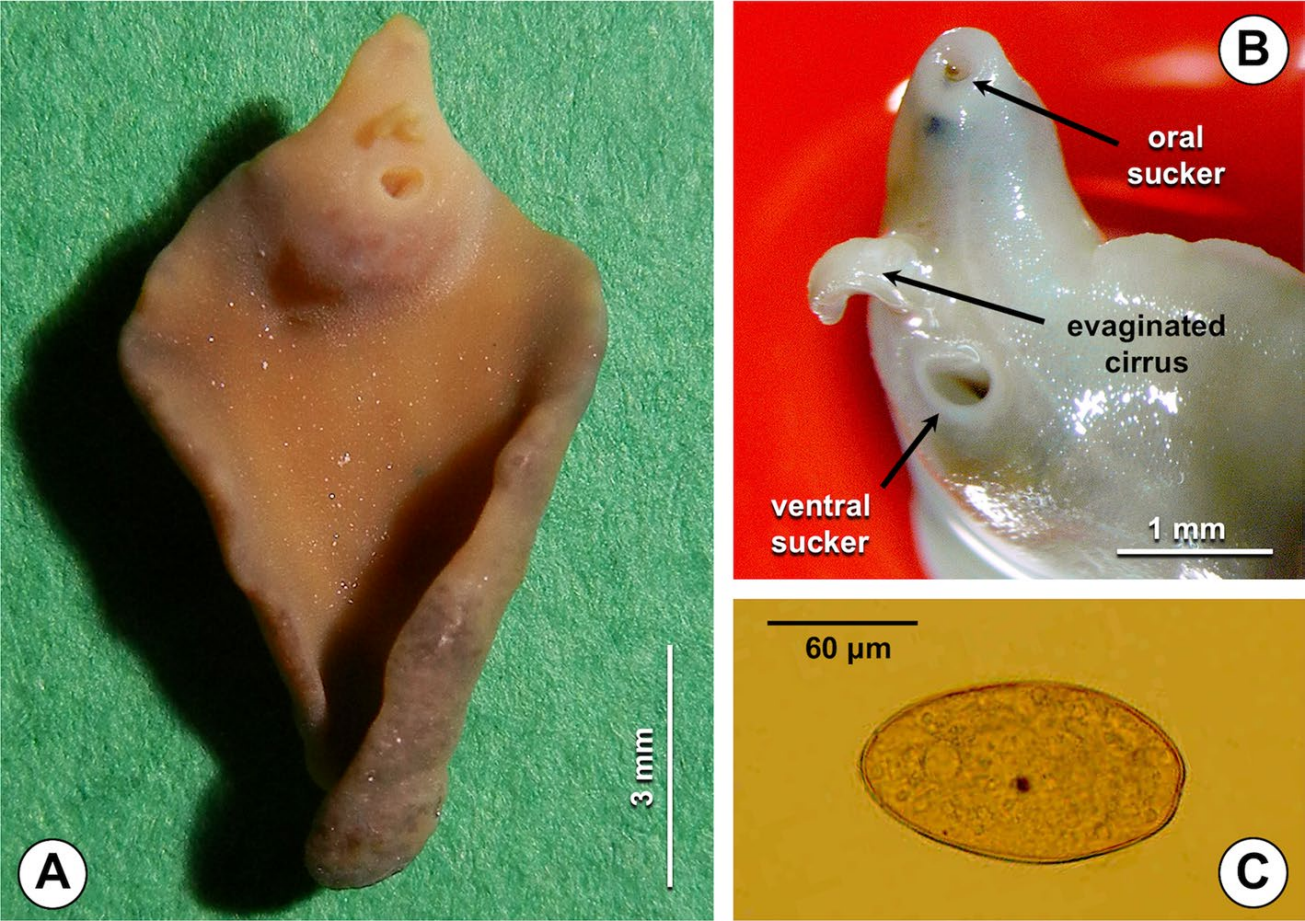
Fasciolid specimen surgically found inside the gallbladder of the patient from Birjand during cholecystectomy: **A** whole specimen in ventral view (note cephalic cone, shoulders, and dark-reddish gravid uterine area below the ventral sucker); **B** anterior extremity showing the two suckers and the evaginated cirrus; **C** egg

Measurements (in mm) obtained in the fasciolid specimen surgically extracted from the patient (Fig. 2) were as follows: body length = 10.8; maximum body width = 6.1; oral sucker diameter = 0.2; distance between the oral sucker and the ventral sucker = 1.5; distance between the ventral sucker and the posterior end of the body = 9.0; distance between the anterior end of the body and the ventral sucker = 1.7; cone length = 1.5; and cone width = 2.4.

The ratios calculated for the same specimen were: body length over body width = 1.77; body length over the distance between the ventral sucker and the posterior end of the body = 1.20.

Egg measurements were 130.8–133.8 µm in length and 73.7–86.1 µm in maximum width.

### Molecular Characterization

The sequencing of the mtDNA *cox*1 gene fragment obtained from the surgical specimen from Birjand provided a 247-bp-long sequence, with a biased AT content (63.73%). The new sequence obtained is available in the Genbank database under accession number LC463032.

The comparison of this *cox*1 fragment with available haplotypes of the 1533-bp-long complete sequence of the *cox*1 gene in genetically “pure” *Fasciola* species from different countries around the world demonstrated that it matches with its partial sequence between positions 803 and 1049. A first nucleotide analysis showed that the *cox*1 fragment from the Birjand specimen matches more with *F. gigantica* than with *F. hepatica*. The BLASTn analysis showed similar results, with a 98–99% similarity with many other fragment sequences of *F. gigantica* isolates from different countries. The most similar proved to be an Elazig isolate of *F. gigantica* from Turkey (unpublished GenBank acc. no. GK12127), from which it differs by only one mutation.

The corresponding comparison alignment showed that a total of 14 variable positions included in this fragment discriminate between genetically “pure” *F. hepatica*, “pure” *F. gigantica*, and the Birjand sequence. Sequence polymorphisms were checked and confirmed by visualization of the raw sequence to assign a mitochondrial profile. Confluent heterozygotic *F. hepatica*/*F. gigantica* sites were detected in the same positions in both primer sequence readings. Among the aforementioned 14 polymorphic sites appearing in the alignment, 7 positions of the Birjand sequence presented double nucleotide composition with *F. gigantica* dominance in 5 of them. In addition, other seven positions showed single nucleotide composition differing from one or other of the “pure” species, including five presenting the same nucleotide as *F. gigantica*, one position with the same nucleotide as in *F. hepatica*, and finally another differing from the two “pure” and therefore representing local variability (Table 1).

**Table 1:** Positions of the mtDNA *cox*1 gene sequence including nucleotides allowing for the differentiation of “pure” *Fasciola hepatica*, “pure” *F. gigantica* and the *Fasciola* specimen surgically obtained from the patient from Birjand, Iran

Species	Length	Differing positions													
	bp	876	927	930	939	945	951	966	984	1002	1009	1011	1014	1018	1026
“Pure” *F. hepatica*	1533	G	G or A	T	C	C	G	G	A	G	A	A	G	A	A
“Pure”* F. gigantica*	1533	A	T or C	G	T	T	A	T	T	A	G	T	T	G	G
*Fasciola* from Iranian patient	247	A/G	T	G/T	T/C	T/C	A/G	T/G	T	G	G/A	T	C	G	G

Number of positions correspond to the complete 1533-bp-long *cox*1 gene sequence. Heterozygotic positions show the two nucleotides detected separated by a slash, the one before the slash being the dominant. The “pure” species show variation in position 927

## Discussion

### Fasciolid Classification and Geographical Analysis

The morphology of the specimen obtained showed pronounced shoulders and non-parallel lateral body walls, initially suggesting its belonging to the species *F. hepatica*. For the morphometric analysis, it should be considered that fasciolids present different morphometric characteristics depending on the host species, humans included, even inside the same endemic area [13]. Given that morphometric variability studies on fasciolid adults infecting humans are lacking for evident reasons, comparisons were made with fasciolid morphometrics found in sheep, goat, cattle, and buffalo in the same country of Iran [16]. These comparisons showed that the measurements obtained in the surgically obtained fluke fit well within the extreme values of fasciolids in cattle.

Three measurements obtained in the surgical specimen perfectly fit in the characteristics of *F. hepatica* according to standards of “pure” species infecting cattle [14], namely: (i) the distance between the ventral sucker and the posterior end of the body, (ii) the ratio of body length over body width, and (iii) the ratio of body length over the distance between the ventral sucker and the posterior end of the body. None of the other body measurements obtained are useful for species differentiation.

The measurements of the fasciolid eggs found in the Birjand patient fit the standard range of *F. hepatica* in areas where *F. gigantica* is absent (100.6–162.2/65.9–104.6 µm) and also that of *F. hepatica*-like flukes in areas where the two species overlap (106.5–171.5/63.9–95.4 µm) [8]. This means that egg size agrees with the morphological characteristics and morphometry of the adult specimen.

Opposite to this, the molecular analysis confirms that the mtDNA profile of the *cox*1 gene fragment from the fasciolid specimen surgically obtained in Birjand corresponds to a fasciolid form between genetically “pure” *F. gigantica* and “pure” *F. hepatica* forms [1]. The detailed nucleotide position analysis shows, moreover, that the 247-bp-long *cox*1 sequence is markedly closer to *F. gigantica* than to *F. hepatica*. Summing up, the Birjand specimen could be phenotypically cataloged as a *F. hepatica*-like form, whereas it may be molecularly considered a *F. gigantica*-like form. The detailed nucleotide analysis indicates the presence of the *cox*1 gene sequences of the two species in the fasciolid specimen, with evident majority of copies of the one of *F. gigantica*.

Up to the present, an intermediate genotype was only reported once in Iran, namely thanks to only one mutation in a ribosomal DNA 18S fragment found in fasciolid material obtained in sheep and cattle from a slaughterhouse of the province of Fars, a southern region of Iran (Fig. 1) [22]. Worth emphasizing is also the existence of a similar *cox*1 sequence differing by only one mutation in the locality of Elazig, in central-eastern Turkey (Fig. 1), where *F. hepatica* was molecularly detected in sheep by ribosomal DNA ITS-2 sequencing [23].

Phenotypic intermediate forms have been reported from livestock in Guilan province [15, 16], and also in latitudes similar to those of the South Khorasan province in neighboring countries such as Pakistan (Fig. 1) [17] or southern Asian countries such as Bangladesh [24]. Interestingly, a similar human case of *Fasciola gigantica-like* infection has recently been reported from Nepal [25]. The infection of humans by such fasciolids has already been reported in South East Asia [26].

It should be highlighted that this is the first time that fascioliasis is reported from the South Khorasan province. So far, in the eastern part of Iran, fascioliasis had been never reported in humans and findings in livestock only concerned Northern Khorasan [27, 28] and Razavi Khorasan [29], two provinces located neighboring Turkmenistan and northward from South Khorasan (Fig. 1). These eastern provinces of Iran are, thus, far from the northwestern and western provinces where human fascioliasis has been repeatedly reported.

### Misdiagnosing Fascioliasis Presentations

Diagnostic problems posed by fascioliasis and consequences of resulting delays in reaching the correct diagnosis due to lack of awareness on the disease have been highlighted by physicians in areas where human infection has been never or only very rarely found or reported. Such situations have been reported in countries as different as USA [30], Switzerland [31], Turkey [32], and India [33, 34].

Non-invasive image techniques may considerably contribute to diagnosis, characterization, and management of pathogenic processes affecting the liver. The main inputs of these techniques are in early detection, helping to establish a definitive diagnosis, and guiding appropriate interventions or surgery. Image techniques have been increasingly available in recent years, including small clinics and health centers in villages of rural areas and peripheric suburbs of cities, where patients inhabiting rural areas are mostly diagnosed. Thus, these techniques may even be routinely applied for liver scanning when patients are presenting with symptoms suggesting liver affection. Helpful image techniques applied to fascioliasis include radiology, radioisotope scanning, ultrasound (US), computed tomography (CT), and magnetic resonance (MR) [12]. However, the images furnished by these techniques may be confusing, their correct interpretation needs long expertise, and may lead to a misdiagnosis sometimes ending in an unnecessary surgical intervention. Several cases have been reported in which fascioliasis diagnosis was only reached with final surgery [35–41].

There are many patient affections in which these techniques may furnish liver images posing interpretation difficulties leading to misdiagnosis.

Biliary colic, jaundice or even pancreatitis indicating bile circulation blockage effects suggests lithiasis. Indeed, in human fascioliasis, stones are originated which may be single, although they are usually small and multiple and have been described as typical pigment stones (PS) [42]. It has been experimentally demonstrated that gallstone presence increases with infection time and is strongly associated with the number of flukes [43]. Indeed, lithiasis suspicion underlies the very long delays in reaching the correct diagnosis and the high number of surgical interventions in fascioliasis patients recorded in Argentina [44].

Similar images may also be obtained in other infections by other infectious organisms. Among helminthiases, other trematodiases such as schistosomiasis, opisthorchiasis, and clonorchiasis, and mainly alveolar echinococcosis, are parasitic diseases to be considered in the differential diagnosis when applying these techniques depending on their respective endemic areas [45]. Hepatic ameobiasis may be distinguished by a homogenous abscess and a high count of granulocytes, and unilocular hydatidosis by a limited and homogenous liquid cyst.

Bacterial infections may also give rise to pyogenic liver abscesses to be considered. Indeed, bacteriological bile culture in fascioliasis infection revealed viable bacteria, including *Escherichia coli* in 45% of cases, *Enterococcus faecalis* in 45%, and *Klebsiella pneumoniae* in 10%. The analyses suggested an association between bacterobilia and the duration of fasciolid infection, intensity of fasciolid infection, and liver damage, and indicated that the obstruction in advanced chronic fascioliasis may be also related to biliary sepsis [46]. In fact, *E. coli* has been reported as the most common isolated microorganism, although recent data show that *K. pneumoniae* is the most common pathogen in pyogenic liver abscesses [47].

Abnormal subcapsular processes may be detected by image techniques. CT has been repeatedly used for the detection of subcapsular hemorrhages, hepatic necrosis, and abscesses due to the penetration of metacercariae through the liver capsule [48–50]. Such presentations are caused by migrating juvenile flukes in the acute phase of the disease. Migrating juvenile flukes may also underlie apparently malignant formations in other organs, such as peritoneum [51], ovary [52], and colon [53].

A variety of parenchymal lesions may be caused by *Fasciola* during the long chronic phase and be detected by image techniques. Several of these techniques are even able to detect the live movement of the worms within the dilated ducts and gallbladder. Such lesions include nodular lesions, abscesses, cystic cavities, hemorrhages, necrosis, eosinophilic granulomas, fibrosis, and even large-sized and multiloculated formations described as liver masses [54].

Images showing abnormal bile ducts or liver lesions may sometimes be confusing and be misdiagnosed as malignant processes. Examples are neoplastic formations, intrahepatic cholangiocarcinoma, biliary cyst adenocarcinoma, metastatic disease, and locally advanced gallbladder carcinoma. In these fascioliasis presentations, misdiagnosis may lead to unnecessary, complex surgical intervention to treat a suspected tumor disease. This problem has been highlighted in many recent reports [55–61].

### Avoiding Unnecessary Liver Surgery Interventions

Reaching a correct diagnosis in the aforementioned confusing fascioliasis presentations is essential not only to avoid procedure delays and unnecessary surgery, but also because a correct drug treatment may be sufficient. It has been observed that such lesions decreased in number and size and finally disappeared or calcified after successful treatment, whereas they increased or remained unchanged when treatment failed [62].

Before deciding to go for surgical intervention, the following analyses and procedures should be made.

(A) Eosinophilia is one of the most helpful markers for human fascioliasis. However, it should be considered that in areas where other helminths usually infect humans, it may be misleading. Moreover, similarly as observed in the patient from Birjand, there have been several reports about human infection by *Fasciola* in patients showing no eosinophilia (see review in [7]), even in cases presenting with lesions in the chronic phase posing the aforementioned diagnosis problems [55].
(B) Modifications of the liver function tests in both acute and chronic phases [6] may also help. Complete blood counts may be of additional value, given that fascioliasis is a cause of anemia [63] and immunosuppression [64]. Anyway, alterations in biochemical markers and hematological parameters may be interpreted with caution, because they may decline in old chronic infections by only very few flukes, mainly when restricted to the gallbladder, and also be caused by other liver infections.
(C) Anamnesis may be crucial when the patient furnishes information on suspicious habits, behavior, and living characteristics. Two aspects should be given priority, namely the place of residence and human infection sources [65, 66]. Living in or visiting a rural area in which there is livestock infected by *Fasciola*, and working or being linked in a way or other with agriculture, farming, livestock management or husbandry, are aspects clearly suggesting liver fluke infection risk [66].
(D) Similarly, physicians should be aware of and updated about the present knowledge on human fascioliasis infection sources. Infection occurs by ingestion of metacercariae. A high diversity of infection sources has recently been shown, including foods, water, and combinations of both. Freshwater wild plant ingestion is the main source, with watercress and secondarily other vegetables. Freshwater cultivated plants, terrestrial wild plants, and terrestrial cultivated plants also appear involved. Urban infections are due to the sale of vegetables in uncontrolled markets and traditional local dishes made from sylvatic plants. Other sources comprise drinking of contaminated water, beverages, juices, and soups, and also washing of vegetables, fruits, tubercles, and kitchen utensils with contaminated water. Religious traditions underlie infection by the ingestion of raw ruminant liver. All this becomes crucial in anamnesis interpretation and may be of great help in guiding physicians to a correct diagnosis [65].
(E) The main tools for the confirmation of *Fasciola* infection in humans are stool and serological techniques, which have been pronouncedly improved for patient diagnosis in recent years [18]. Serological methods using commercial kits may be helpful in cases of negative stool examination. In clinical practice, in non-endemic countries, the diagnosis is sometimes misdiagnosed as liver cancer in front of very alarming CT scan or MRI images; serology is helpful in such cases, together with fever, pain, eosinophilia, and jaundice [67]. Present availabilities of fascioliasis diagnostic techniques have recently been reviewed [18], furnishing a new baseline to help and guide physicians in their patient diagnosis work concerning: advantages and weaknesses of the tests, sample management, egg differentiation, qualitative and quantitative diagnosis, antibody and antigen detection, post-treatment monitoring, and post-control surveillance. Difficulties posed by fascioliasis diagnosis in patients are emphasized in front of: the different infection phases and parasite migration capacities, clinical heterogeneity, immunological complexity, and different epidemiological situations and transmission patterns. The lack of a diagnostic technique covering all needs and situations, and the advisability for a combined use of a coprological and a serological technique, are highlighted [18].
(F) In the diagnosis of individual patients, it is important to consider that the acute and the subsequent chronic phases of fascioliasis require different diagnostic approaches. During the initial acute phase, direct coprological analyses are useless because fasciolid eggs cannot be found in patient stools as no egg-producing adult flukes have been yet originated in the biliary canals. Therefore, for the diagnosis of this acute phase, serological techniques are needed which pose other kinds of problems concerning sensitivity and specificity, besides the impossibility to differentiate between *F. hepatica* and *F. gigantica* [18]. In the chronic phase, a diagnostic confirmation may be obtained by fasciolid egg finding in the patient stools or duodenal aspirate, which moreover may allow for *Fasciola* species differentiation and a quantitative analysis for burden estimation. The latter is needed to define the treatment dose in patients shedding more than 400 eggs per gram [68]. However, eggs may not be produced in humans in given areas, or produced in very low numbers that may be overlooked [18]. A coproantigen-detection test [68], combined with a new diluent developed for the preservation of *Fasciola* coproantigens [69], has been one of the most useful recent progresses, despite its uselessness for fasciolid species differentiation and fluke burden evaluation.
(G) Another way for *Fasciola* infection confirmation is by cholangiography using endoscopic retrograde cholangiopancreatography (ERCP). In biliary obstruction, ERCP and sphincterotomy have been used to extract parasites from the biliary tree by balloon or basket [12]. ERCP is, therefore, considered the gold standard for bile duct imaging, for the rapid correct diagnosis, and endoscopic treatment by direct clearance of the bile ducts in patients in the chronic phase [70]. This technique has been also successfully used in fascioliasis presentations mimicking cholangiocarcinoma, and thus avoiding surgery [71].
(H) An important aspect to be considered by surgeons is the convenience of fixing and preserving the flukes extracted in alcohol instead of in formalin, to facilitate subsequent appropriate DNA sequencing methods enabling for a specific diagnosis of the causal agent.
(I) Histological examination of liver biopsy materials may reveal egg granulomas or fluke sections which may also help in diagnosis confirmation. Image techniques may greatly help in guiding the successful obtaining of these samples.

At present, triclabendazole is the drug of choice for fascioliasis. This drug has the advantage of being the only one efficient against both tissue-migrating juvenile flukes and adult flukes in biliary canals and gallbladder [72]. Triclabendazole has moreover proved to be useful for the treatment of liver fluke infection in very small children aged only a few months [73]. Another drug as nitazoxanide may be an alternative in non-human endemic areas where lower human infection probabilities should usually underlie very low burdens in humans, although only to treat in the chronic phase [74]. This does not mean, however, that cholecystectomy, considering the pathogenetic effects of flukes on the organ, may be mandatory in advanced cases of cholecystitis and obstructive jaundice [70].

## Concluding Remarks

### Fascioliasis Case Detection and Fluke Classification in Non-endemic Areas

The reported unexpected finding of a fasciolid fluke inside the gallbladder of a patient during cholecystectomy performed due to obstructive lithiasis suspicion was related to the fact that the patient was living in an area where no previous information about local human and/or animal fascioliasis was available.

The morphometric phenotyping and DNA sequencing genotyping proved the surgically obtained fluke to be an intermediate fasciolid form. This is the first report of such a liver fluke infecting a human in Near East Asia. This finding suggests that hybridization phenomena may be also linked to the increasing human infection risks in areas of overlap of *F. hepatica* and *F. gigantica* throughout Asia and Africa [1].

An initial geographical overlap of both *Fasciola* species in western Asia has recently been established to have occurred before the Neolithic throughout the so-called “Fertile Crescent” including from Anatolia (present-day Turkey) and Iran [75], from where the two fasciolids expanded westward throughout Europe and eastward throughout Asia [76]. The spreading capacities of hybrids higher than those of pure species are well known in animals. Indeed, the involvement of *Fasciola* hybrids in human fascioliasis has already been reported in South East Asia [26] and has recently been verified to be ongoing in southern Asia [77].

Although there is a total lack of studies on fascioliasis in the South Khorasan Province, it shall be considered that Birjand was linked to the network of caravan routes along many centuries in the past [78]: (i) throughout northern Asia by the Silk Road connecting with China through Samarkand and Kashgar, and (ii) throughout southern Asia via Afghanistan and Pakistan to northern India and Bangladesh by the Grand Trunk Road whether traversing the Kyber Pass or along the secondary route of Alexandria in Aria, both leading down to the old locality of Taxila [76]. The cooler northern Silk Road was appropriate for the spread of *F. hepatica* although *F. gigantica* also took advantage from it, whereas the warmer southern Grand Trunk Road was mainly used by *F. gigantica*. These archeological data and historical records do, therefore, support the coexistence of both *F. gigantica* and *F. hepatica* infecting domestic ruminants, equids, and Old-World camelids used as pack animals in the caravans reaching Birjand in the past. The recent new worldwide baseline furnished by the complete mapping of the evolutionary spreading of fasciolids does hereby prove its usefulness for the understanding of the fascioliasis patient in Birjand [76].

### Avoiding Unnecessary Surgery in Fascioliasis Patients

Climate changes, mainly global warming temperatures and altered rainfall regimes, and global changes, mainly anthropogenic modifications of the environment, human movements, animal exportation and importation, and an increasing livestock demand by the growing human populations in many parts of the world, will influence the fascioliasis transmission rates in the near future. This will imply increasing human infection risks in areas where this disease is nowadays neglected in humans [12]. These areas are indeed those where physicians and health personnel are less aware of fascioliasis. A consequence may be increasing misdiagnosis in patients in cases of infection presentations and use of exploration techniques potentially leading to confusion. Lithiasis, hepatic colic, jaundice, and obstruction, on one side, and apparently malignant liver processes of different types, on the other side, besides image techniques of insufficient resolution in several situations, may underlie correct diagnostic delays and unnecessary surgical interventions.

Increasing awareness of human infection risk by *Fasciola* and of the way to avoid misdiagnosis and unnecessary surgery becomes a need. The aforementioned list of analyses and procedures for the correct diagnosis of fascioliasis patients furnish an important addition to the control baseline of this worldwide distributed disease, whose highly complex transmission characteristics and heterogeneous epidemiology give rise to individual infections leading to patients attending hospitals and health centers [79].
